# Correction: Evaluating community pharmacists’ involvement in patient counselling and health education in Nairobi, Kenya: A cross-sectional study

**DOI:** 10.1371/journal.pone.0349419

**Published:** 2026-05-14

**Authors:** Seraphine Wanjiro Manjari, Ermias Mergia Terefe

The second author’s name is incorrect. The correct name is: Ermias Mergia Terefe. The correct citation is: Manjari SW, Terefe EM (2026) Evaluating community pharmacists’ involvement in patient counselling and health education in Nairobi, Kenya: A cross-sectional study. PLoS One 21(1): e0322829. https://doi.org/10.1371/journal.pone.0322829.
