# Supplementary material for: Evaluating community pharmacists’ involvement in patient counselling and health education in Nairobi, Kenya: A cross-sectional study
Source: PLoS One. 2026 Jan 22;21(1):e0322829. doi: 10.1371/journal.pone.0322829 (PMC12826495; doi:10.1371/journal.pone.0322829)
Supplement: Attachment — Submitted filename: Kenya community pharmacy PlosOne.docx [file pone.0322829.s001.docx]

**PLOS ONE**

**Evaluating community pharmacists' involvement in patient counselling and health education in Nairobi, Kenya: A cross-sectional study.**

Thank you for the opportunity to review this important manuscript. The authors tackle a relevant issue which is the discrepancy between self-reported and actual practices of community pharmacists in Nairobi regarding patient counselling and health education. The use of both self-report surveys and simulated patient methodology is commendable and aligns well with international best practices. However, there are several key concerns related to methodology, writing clarity, contextual framing, and depth of discussion. Unfortunately, I regret to inform you that after careful consideration, I have decided to reject your manuscript for the following reasons:

- Inconsistent methodological framing. You describe using a “qualitative descriptive research design,” yet the methodology (structured questionnaires and numerical comparison of self-report vs. observed behaviour) is entirely quantitative. This undermines credibility and shows a lack of understanding of research paradigms.
- Qualitative descriptive research explores how and why perceptions and phenomena exist. In the case of your study, this is not a qualitative design. You started off by reviewing by assessing pharmacists’ self-reported perception of their practices before evaluating their performance with simulated scores.
- There is no mention of validation or piloting for the structured questionnaires or the simulated patient checklists—no psychometric testing, no reference to adaptation from prior tools, and no evidence of reliability checks (e.g., inter-rater reliability among SPs). This is very important as we cannot be sure of the reliability of your findings. While you do mention a pilot study was conducted, it’s not clear who this pilot was on and how it was done.
- Simulated patient method section lacks rigor. While SP methods are appropriate and valuable, this implementation is methodologically inadequate as there is no detail on how SPs were trained to ensure standardization, no verification of inter-rater agreement across scenarios, and it is unclear whether pharmacies knew about the study timeframe (risk of contamination or bias). If pharmacists completed the self-assessment survey, the risk of performance bias is relatively high.
- Your analysis is largely superficial as you rely solely on descriptive statistics without reporting any inferential statistics, adjusting for demographic variables, or exploring associations between self-reported and observed practices.
- Your data interpretation is overstated. The claims in the discussion far exceed the strength of the underlying data. They generalize poor performance without addressing why it may be happening, nor do they explore confounders or local context adequately. Moreover, I have issues with your discussion as it is a repletion of the results without providing any real substance or context. This discussion doesn’t answer any new questions or unclear gaps in the literature, particularly in developing countries.
- Overall, I’m not overly convinced on the premise of your study or how in its current form it adds value to the literature. The topic of pharmacy practice in developing countries is crucial but your paper has superficial analysis and with major flaws in its study design, will be very difficult to extrapolate to international readers from PlosOne.

Please note I have provided you with in depth comments for each of the relevant sections of your manuscript to consider as you adjust to submit to another journal:

**Abstract:**

- Avoid vague terms like “significant discrepancies” without stating what statistical tests or thresholds were used or how this was determined.
- You should clearly describe the data collection methods (e.g., “structured questionnaires and simulated patient scenarios”) and specify how the comparisons were made. This should be clear from the abstract.
- Consider adding a line stating the total number of simulated patient visits conducted for transparency.

**Introduction:**

- The introduction overuses general statements that don’t add much beyond what is already known globally about community pharmacy roles. For example, in page 3 lines 67 – 71, you make a very general statement about community pharmacists which is entirely inaccurate in many parts of the world (not to mention that this not even referenced). Instead, focus your introduction more on the Kenyan or East African context early on, and incorporate a clearer rationale for why simulated patient methodology is particularly valuable in LMICs where self-report may be unreliable.
- Lines 72 – 75: If you are not several studies, you need to show more than one reference. This is not accurate scientific writing.
- Your objective is relatively unclear. You should end with a precise research aim and rationale for using both survey and simulated patient methods.

**Methods:**

- There is no description of how simulated patients were trained or whether they piloted the cases to ensure inter-rater reliability. What training, if any, was done to ensure standardization of the simulated encounters?
- No information is given on how the simulated patient data were coded or analyzed beyond simple frequencies. Did multiple raters score the pharmacist performance? Was inter-rater reliability assessed?
- A major limitation is the lack of detail about the questionnaires and checklists used. Were these adapted from existing tools? If developed by the authors, was there any validation process?

**Results:**

- You make strong claims about patient satisfaction being low, yet satisfaction scores are only described in vague terms (e.g., “most simulated patients rated their encounters poorly”). Please provide mean/median values and standard deviations or ranges.
- There is a lack of clarity on why the simulated patient scores of the pharmacists’ performance were based a scale of 0 – 20. How accurate are the assessment of these student SPs and who checked over them?

**Discussion:**

- The discussion appropriately highlights the discrepancy between reported and actual practice, but it remains largely descriptive. It would benefit from deeper reflection on why these gaps exist (e.g., training, workload, commercial pressures) and specific policy or curricular interventions that could address the issues.
- The discussion currently lacks international framing. You mention comparisons to studies in Saudi Arabia and Ethiopia, but broader international relevance is needed. How might this study inform similar contexts in Sub-Saharan Africa or elsewhere?
- Several limitations are not adequately addressed such as:
  - Potential Hawthorne effect if pharmacists suspected the simulated visits.
  - Lack of triangulation or qualitative follow-up to understand of why you believe pharmacists underperform.
  - Absence of statistical comparisons weakens the rigor of observed differences.
