# Supplementary material for: Evaluating community pharmacists’ involvement in patient counselling and health education in Nairobi, Kenya: A cross-sectional study
Source: PLoS One. 2026 Jan 22;21(1):e0322829. doi: 10.1371/journal.pone.0322829 (PMC12826495; doi:10.1371/journal.pone.0322829)
Supplement: Attachment — Submitted filename: RE-Rebuttal Letter- Point by Point Respnse.docx [file pone.0322829.s004.docx]

**Rebuttal Letter**

**Manuscript Title:** *Evaluating community pharmacists’ involvement in patient counselling and health education in Nairobi, Kenya: A cross-sectional study*

**Authors:** Manjari Seraphine Wanjiru, Ermias Mergia Terefe

**POINT-BY-POINT RESPONSE TO EDITOR & REVIEWERS**

**Response to Academic Editor**

**Comment:**
“Heartfelt thanks… However, a few minor revisions have been suggested in the abstract, introduction and limitation sections.”

**Response:**
We thank the Academic Editor for the positive feedback. We have revised the abstract, introduction, and limitations section to correct methodological wording, incorporate missing citations, and clarify study constraints. All changes have been tracked in the revised manuscript.

**Response to Reviewer #1**

**Comment 1:**

The manuscript inaccurately described the design as “qualitative descriptive” while the study is quantitative cross-sectional.

**Response:**

We thank the reviewer for highlighting this important issue. We confirm that the earlier version of the manuscript contained the term “qualitative descriptive design”, which was indeed incorrect. This has already been fully corrected in the revised manuscript.

The study is now consistently described as:

“a cross-sectional descriptive design incorporating a simulated patient methodology.”

We have re-reviewed the entire manuscript to ensure no residual qualitative terminology remains.

**Comment 2:**

Lack of validation of the survey tool.

**Response:**

We thank the reviewer for this important observation. We have strengthened the description of the survey tool development and validation process to enhance transparency and methodological clarity.

Specifically, we have revised the **Survey Instrumentation and Pilot Testing** subsection to clearly state that:

- Both the questionnaire and the simulated patient (SP) checklist were **adapted from previously validated instruments** used in comparable settings.
- The tools underwent **pilot testing** in six community pharmacies to refine item clarity, contextual appropriateness, and feasibility.
- **Inter-rater reliability** for the SP checklist was assessed and demonstrated strong agreement (Cohen’s kappa = 0.82).
- **Formal internal consistency testing** (e.g., Cronbach’s alpha) was **not performed**, as the pilot sample size was too small to support meaningful psychometric analysis.
- Additionally, although the questionnaire was adapted from validated tools, **no further psychometric validation** (such as internal consistency analysis) was conducted.

To fully acknowledge this limitation, we have added the following statement to the **Limitations** section:

“Although the tools were adapted from validated instruments and pilot tested, formal psychometric validation—such as internal consistency testing (e.g., Cronbach’s alpha)—was not performed, which may limit the overall measurement robustness.”

These revisions directly address the reviewer’s comment and improve the methodological transparency of the manuscript.

**Comment 3:**

Absence of inferential analysis.

**Response:**
We thank the reviewer for this observation. We agree that the use of descriptive statistics alone limits the depth of analysis, particularly regarding potential associations between pharmacist characteristics and counselling behaviours. To address this, we have explicitly added this point to the Limitations section of the manuscript.

The following sentence has been inserted:

“This study relied solely on descriptive statistics, which limited the ability to examine associations between pharmacist characteristics and counselling practices. Future studies with larger datasets should incorporate inferential analyses to explore these relationships more comprehensively.”

This revision acknowledges the limitation clearly and outlines how future research can address it.

**Response to Reviewer #2**

**Comment 1:**

“You mentioned studies from neighboring countries but did not cite Ethiopia and Uganda.”

**Response:**
We thank the reviewer for this helpful observation. We have now addressed this by adding relevant studies from both Ethiopia and Uganda to strengthen the regional context in the Introduction. Specifically, we incorporated citations from recent and high‐quality research examining community pharmacy practice, counselling behavior, and regulatory adherence in these countries.

The following studies have been added:

- **Ethiopia:**
  - Surur et al. (2017) – Self-reported vs. actual involvement of community pharmacists in counselling. (PMCID: PMC5386626)
- **Uganda:**
  - Ocan et al. (2014) – Patterns and predictors of self-medication and pharmacy-related practices in Uganda. (PMID: 38131171)

These citations have now been integrated into the paragraph discussing regional evidence on pharmacist counselling and health promotion, ensuring the manuscript reflects broader East African findings.

We appreciate the reviewer’s guidance, which has improved the completeness and contextual strength of the Introduction.

**Comment 2:**

“Ensure simulated patient is included in the study design mentioned in the abstract.”

**Response:**
We thank the reviewer for this important clarification. We have revised the Methods section of the Abstract to explicitly include the simulated patient component of the study design. The sentence now reads:

“This study employed a cross-sectional descriptive design incorporating both structured questionnaires and a simulated patient (SP) methodology to compare self-reported and observed counselling practices.”

This revision ensures that the simulated patient approach is clearly and accurately reflected in the Abstract, consistent with the detailed methodology described in the main manuscript.

**Comment 3:**

“Discuss the lower sample size as a limitation.”

**Response:**
We thank the reviewer for this valuable observation. We have now explicitly acknowledged the lower-than-estimated sample size as a study limitation. Although the calculated minimum sample size was 92 pharmacies, only 60 were sampled and 48 pharmacists completed the study due to logistical and operational constraints.

To address this comment, we have added the following statement to the Limitations section:

“The achieved sample size (48 pharmacists) was lower than the calculated minimum required sample of 92, primarily due to logistical challenges. This reduced sample size may limit the generalizability of the findings.”

This revision ensures that the limitation is clearly addressed and consistent with the reviewer’s recommendation.
